# A Rare Cause of Chest Pain

**DOI:** 10.7759/cureus.2942

**Published:** 2018-07-08

**Authors:** Saad Saleem

**Affiliations:** 1 Internal Medicine, Mercy St. Vincent Medical Center, Toledo, USA

**Keywords:** foreign body ingestion, sharp pointed bone, mentally health adult, chest pain

## Abstract

Foreign body ingestion occurs mostly among children and mentally impaired adults. We present a case of an esophageal foreign body in a mentally healthy adult presenting with chest pain. The early recognition and treatment of a sharp, pointed foreign body are imperative for appropriate treatment, which can prevent possible complications. The esophageal foreign body should be considered a possible cause of non-cardiac chest pain in the emergency department.

## Introduction

Foreign body ingestion occurs primarily among children and mentally impaired adults. Although it is less common among adults, it can still occur. Food impaction above a pre-existing esophageal stricture is the most common cause of esophageal foreign body obstruction in adults in contrast to children where coins (76 percent in one large series) are the most common [1]. The common foreign bodies typically ingested by adults include fish or chicken bones, medication packaging, dentures, and coins [2]. We discuss a case of a patient presenting with chest pain secondary to foreign body ingestion.

## Case presentation

**Figure 1:**
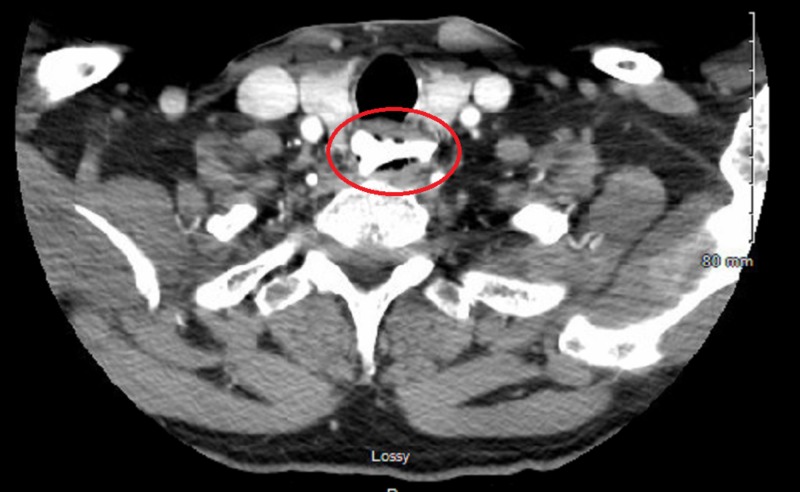
Computed tomography (CT) of neck showing a bone in the esophagus at the level of cervical vertebra 7-thoracic vertebra 1

**Figure 2:**
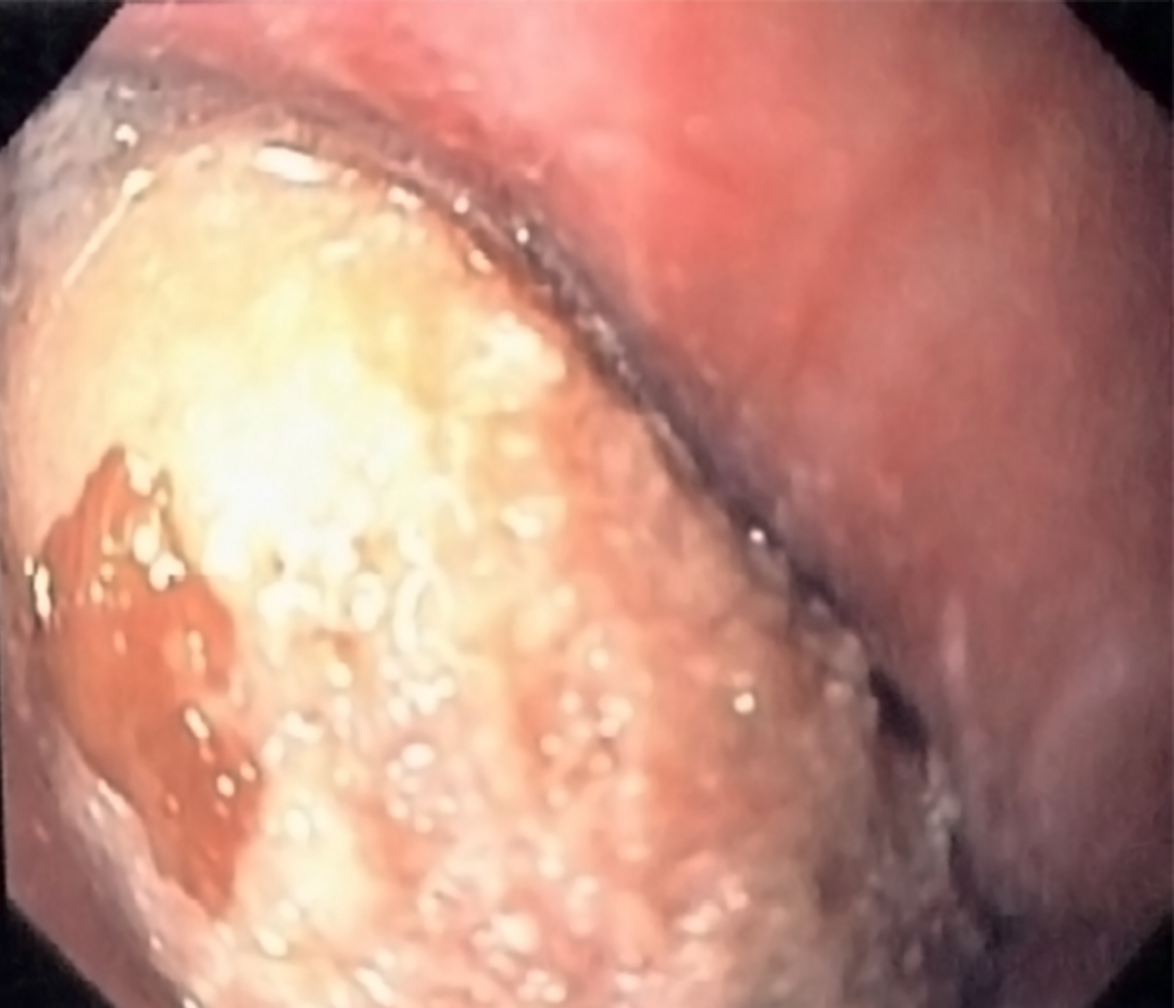
Flexible esophagoscopy showing the piece of bone lodged in the esophagus

**Figure 3:**
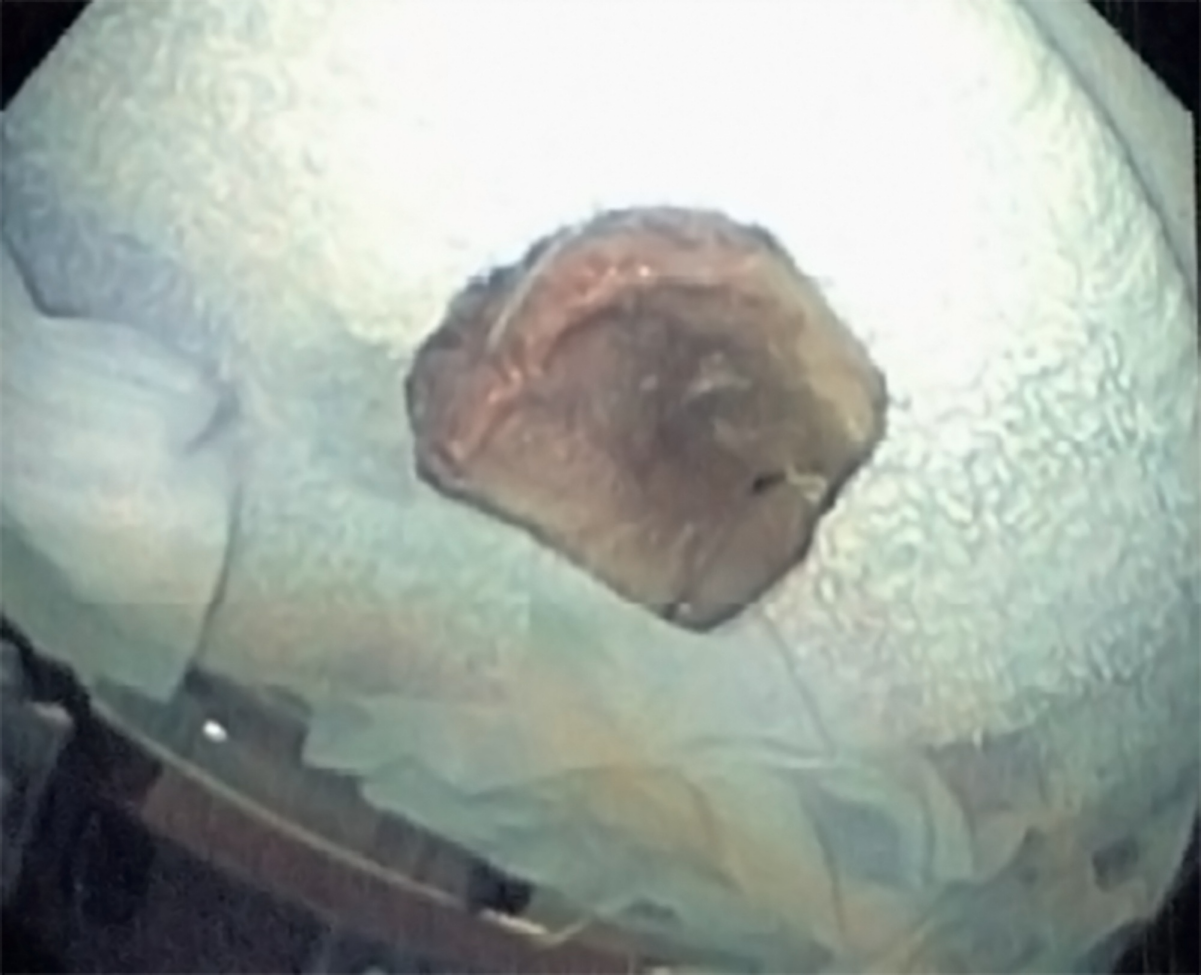
The extracted piece of bone

A 59-year-old male with no significant past medical history presented to the emergency department (ED) with a sudden onset of chest pain and shortness of breath that had begun while he was eating dinner. The physical examination was unremarkable, including the chest examination, except that the patient remained quite anxious. A blood sample analysis was within the normal range, including troponin. The electrocardiogram (EKG) was unremarkable. A plain chest radiograph did not show any abnormality. A computed tomography (CT) scan of the neck revealed a 2.5-cm long rectangular prism-type bone, horizontally lodged in the esophagus at the level of the aortic arch (cervical vertebra 7-thoracic vertebra 1, as shown in Figure 1). Because the patient was symptomatic and the CT neck scan showed the presence of a sharp, pointed bone, esophagogastroduodenoscopy (EGD) was performed under general anesthesia, which involved the retrieval of a piece of bone from the upper esophagus just below the upper esophageal sphincter (Figure 2). The foreign body was removed with the help of queen retrievers (Figure 3). The patient did well after the removal of the foreign body and did not suffer a recurrence of the prior chest pain. He was discharged home on proton pump inhibitors.

## Discussion

A foreign body usually becomes impacted on physiology or pathologic esophageal luminal narrowing. The esophagus has three physiological narrowings, which include the upper esophagus sphincter, the level of the aortic arch, and the diaphragmatic hiatus. Structural (the pre-existing esophagus stricture) or functional (achalasia) abnormalities can increase the risk of foreign body impaction.

The most common presentation of foreign body ingestion is acute dysphagia or the inability to swallow saliva [3]. Complete esophagus obstruction causes the inability to swallow saliva. Some patients present with symptoms of choking, regurgitation of undigested food, or respiratory distress. Chest pain is a common cause of emergency room visits, occurring both due to cardiac and non-cardiac conditions. Esophageal disorders are the most common causes of non-cardiac chest pain [4]. The physician should think of the possibility of an esophageal foreign body when a patient comes to the emergency room for non-cardiac chest pain.

A foreign body is usually detected by plain radiographs, except for fish bones. Computed tomography (CT) neck scans can be helpful if the plain radiograph is negative. They can also be helpful in terms of evaluating the foreign body's position and composition. If the plain x radiograph is negative and the patient is asymptomatic, conservative management is appropriate since more than 80% ingested foreign bodies pass spontaneously without intervention [5].

Endoscopic intervention in the first 24 hours since the time of ingestion should be considered early in adults because delaying intervention may produce more symptomatic esophageal ulcerations with odynophagia [2]. The presence of sharp, pointed objects is a medical emergency due to the risk of esophageal perforation. The object often passes spontaneously through the remainder of the gastrointestinal tract if it enters the stomach but complications have been observed in up to 35% of patients [6]. They should be removed endoscopically, if possible [6]. Surgical consultation is needed if there are signs of esophageal perforation.

The forward-viewing flexible endoscope is recommended for foreign body retrieval, as rigid endoscopes can cause perforation. The patient should be monitored with daily radiographs if the removal of the object is not possible endoscopically. Surgical removal should be considered for foreign bodies that fail to advance over three consecutive days [7].

## Conclusions

The physician should think of the possibility of the presence of an esophageal foreign body when a patient presents to the emergency room with chest pain. It is always better to obtain images of an esophageal foreign body (plain radiograph or computed tomography) for the proper positioning and better visualization of the composition of the foreign body.

## References

[1] Schunk JE, Harrison AM, Corneli HM, Nixon GW (1994). Fluoroscopic foley catheter removal of esophageal foreign bodies in children: experience with 415 episodes. Pediatrics.

[2] Wu WT, Chiu CT, Kuo CJ (2011). Endoscopic management of suspected esophageal foreign body in adults. Dis Esophagus.

[3] Ikenberry SO, Jue TL (2011). Management of ingested foreign bodies and food impactions. Gastrointest Endosc.

[4] Nakshabendi IM, Maldonado ME, Brady PG (2001). Chest pain: overlooked manifestation of unsuspected esophageal foreign body. South Med J.

[5] Carp L (1927). Foreign bodies in the intestine. Ann Surg.

[6] Vizcarrondo FJ, Brady PG, Nord HJ (1983). Foreign bodies of the upper gastrointestinal tract. Gastrointest Endosc.

[7] Eisen GM, Baron TH, Dominitz JA (2002). Guideline for the management of ingested foreign bodies. Gastrointest Endosc.

